# Supplementary material for: The Breadth of the Neutralizing Antibody Response to Original SARS-CoV-2 Infection is Linked to the Presence of Long COVID Symptoms
Source: medRxiv. 2023 Mar 31:2023.03.30.23287923. Preprint. [Version 1] doi: 10.1101/2023.03.30.23287923 (PMC10081395; doi:10.1101/2023.03.30.23287923)
Supplement: 1 [file NIHPP2023.03.30.23287923V1-supplement-1.pdf]

medRxiv preprint doi: https://doi.org/10.1101/2023.03.30.23287923; this version posted March 31, 2023. The copyright holder for this preprint **(which was not certified by peer review)** is the author/funder, who has granted medRxiv a license to display the preprint in perpetuity.
It is made available under a CC-BY 4.0 International license .

SUPPLEMENTARY MATERIALS

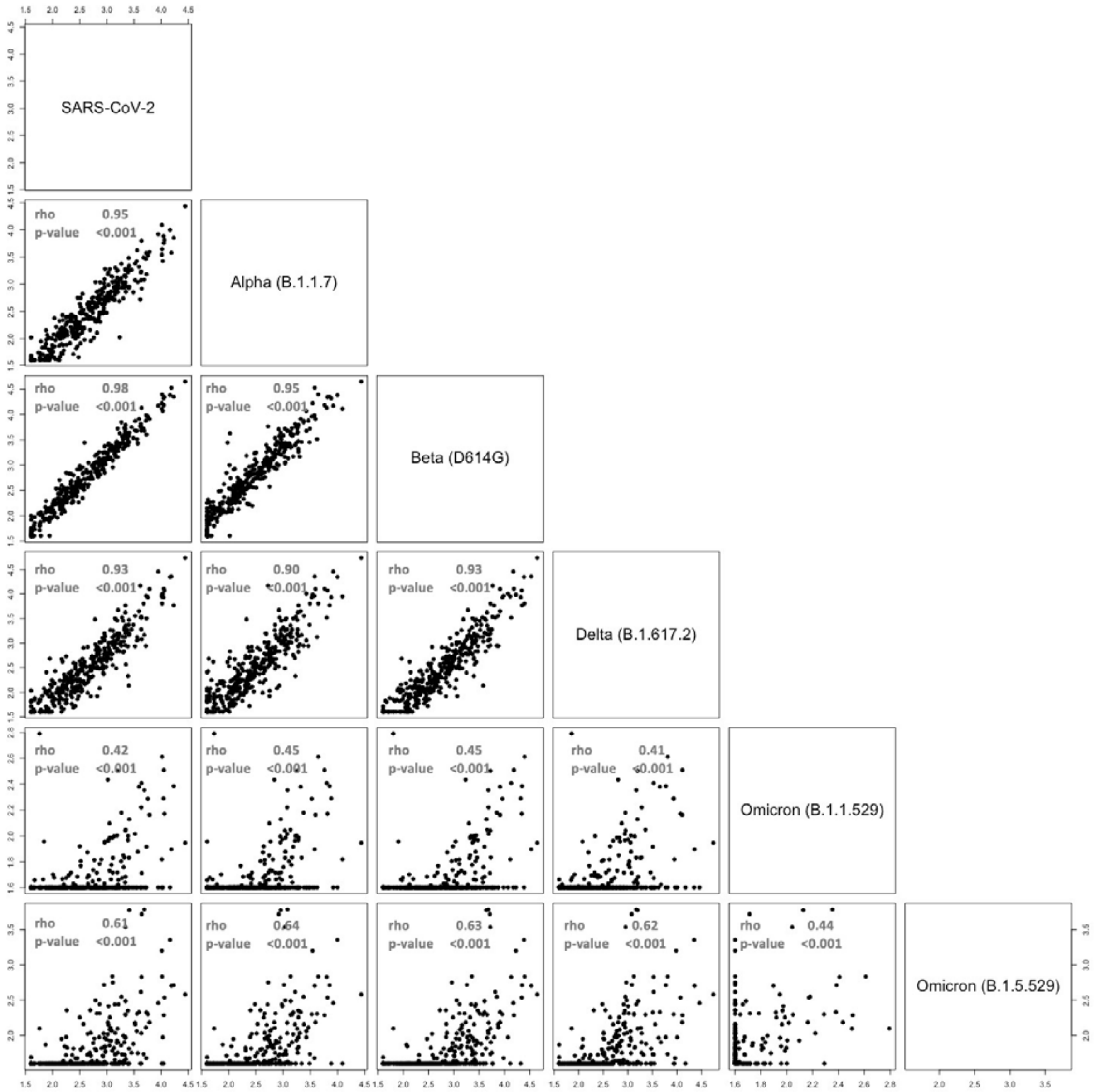

Supplementary Figure 1. Correlation Matrix of Neutralization ID50s For SARS-CoV-2 variants tested. Data across all time points are shown in the matrices. R and P values from non-parametric Spearman rank correlation analyses.

medRxiv preprint doi: https://doi.org/10.1101/2023.03.30.23287923; this version posted March 31, 2023. The copyright holder for this preprint (which was not certified by peer review) is the author/funder, who has granted medRxiv a license to display the preprint in perpetuity.
It is made available under a CC-BY 4.0 International license .

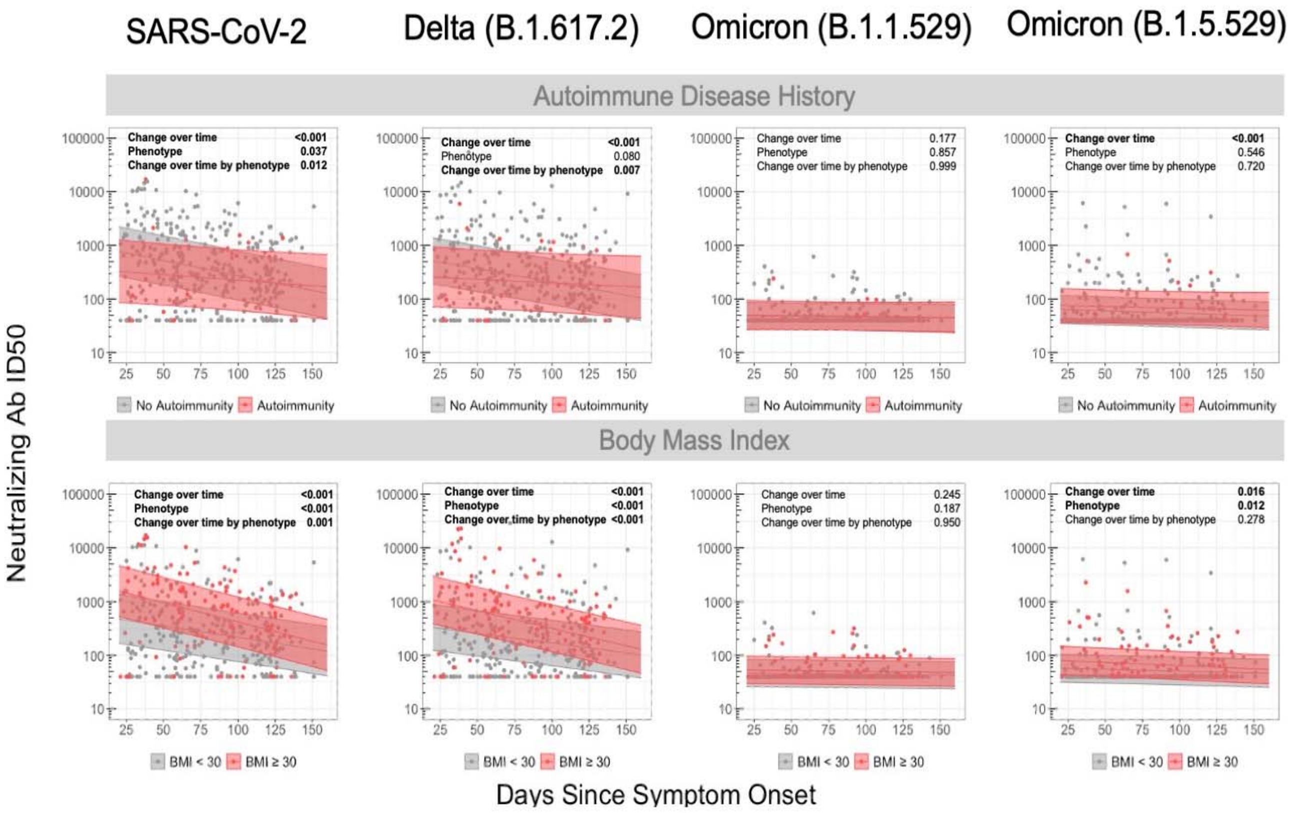

Supplementary Figure 2. Longitudinal analysis of antibody neutralization by clinical factors and SARS-CoV-2 variant. Mixed-linear regression model with two covariates (body mass index ad autoimmune disease history) for different variants: SARS-CoV-2, B.1.617.2, B.1.1.529 and BA.4/5. P-values denote if a significant difference was observed for change in antibody neutralization over time (Change over time), between subgroup (Phenotype*, e.g.,* BMI ≥ 30), and difference in change over time by subgroup (Change over time by phenotype). Shaded region represents 95% confidence intervals around the median line.

medRxiv preprint doi: https://doi.org/10.1101/2023.03.30.23287923; this version posted March 31, 2023. The copyright holder for this preprint (which was not certified by peer review) is the author/funder, who has granted medRxiv a license to display the preprint in perpetuity.
It is made available under a CC-BY 4.0 International license .

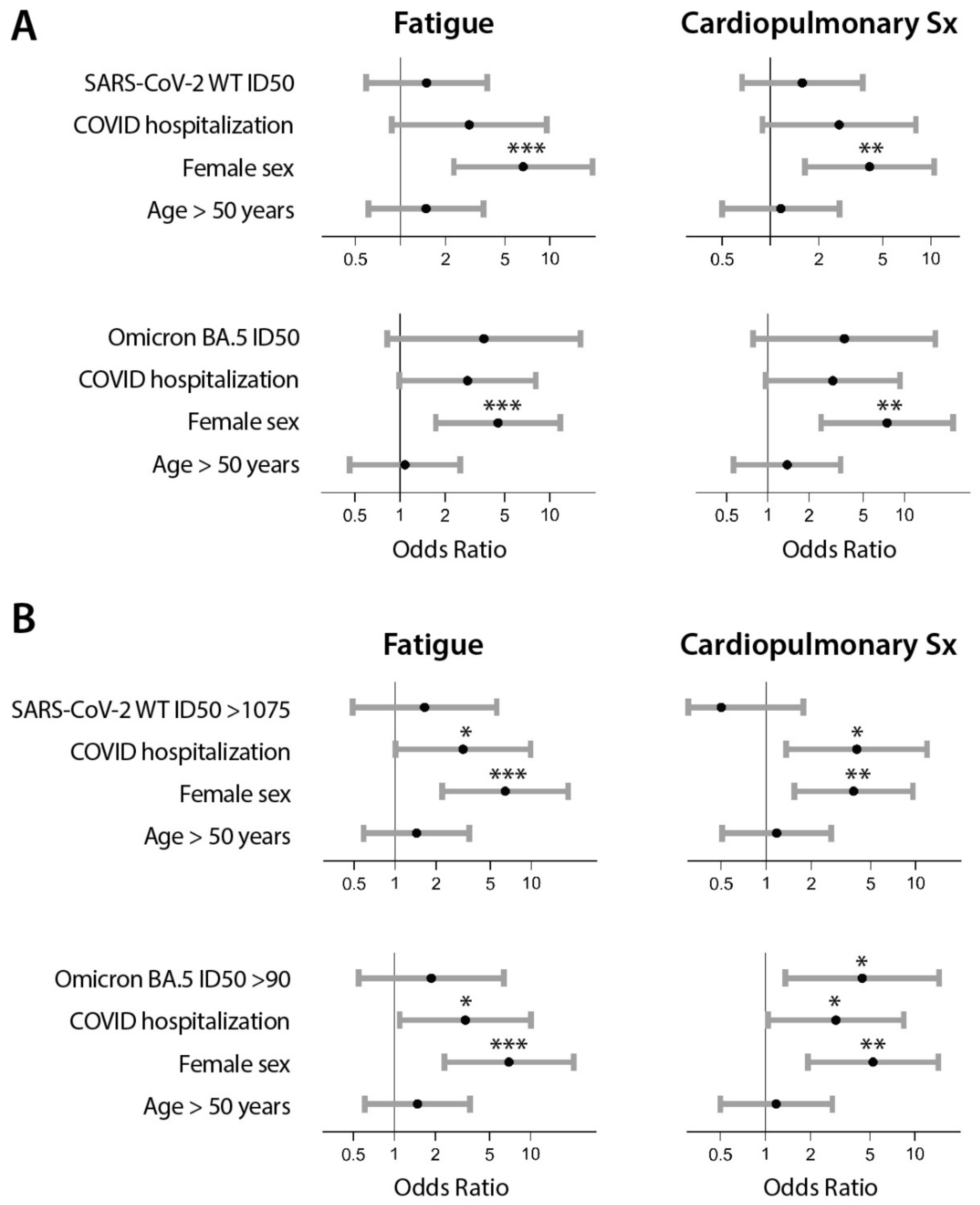

Supplementary Figure 3. Association between SARS-CoV-2 neutralization, hospitalization during acute infection and demographic factors and the odds of experiencing fatigue or cardiopulmonary symptoms approximately four months following acute COVID-19. The top panel shows odds ratios (points) and 95% confidence intervals (bars) for each variable included in logistic regression models using continuous neutralization ID50 values for assays using the original and Omicron BA.5 pseudoviruses (**A**). The bottom panel shows odds ratios and 95% confidence intervals of developing PASC or specific PASC phenotypes for logistic regression models incorporating a binary variable indicating if a sample had a neutralization ID50 in the top 15% of the cohort to either original SARS-CoV02 or Omicron BA.5 pseudoviruses (**B**). *P<0.05, **P<0.01, ***P<0.001 from covariate adjusted logistic regression.

medRxiv preprint doi: https://doi.org/10.1101/2023.03.30.23287923; this version posted March 31, 2023. The copyright holder for this preprint (which was not certified by peer review) is the author/funder, who has granted medRxiv a license to display the preprint in perpetuity.
It is made available under a CC-BY 4.0 International license .

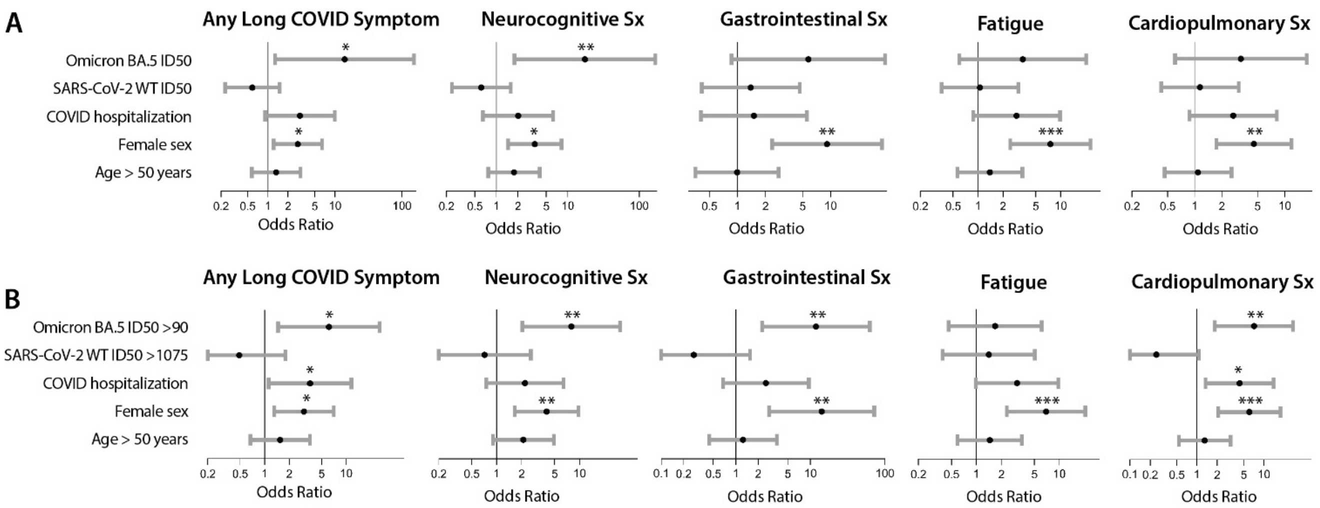

Supplementary Figure 4. Association between SARS-CoV-2 neutralization, hospitalization during acute infection and demographic factors and the odds of experiencing fatigue or cardiopulmonary symptoms approximately four months following acute COVID-19. The top panel shows odds ratios (points) and 95% confidence intervals (bars) for each variable included in logistic regression using continuous neutralization ID50 values for assays using the original and Omicron BA.5 pseudoviruses in the same model (**A**). The bottom panel shows odds ratios and 95% confidence intervals of developing PASC or specific PASC phenotypes for logistic regression incorporating a binary variable indicating if a sample had a neutralization ID50 in the top 15% of the cohort to the original SARS-CoV02 and Omicron BA.5 pseudoviruses (**B**). *P<0.05, **P<0.01, ***P<0.001 from covariate adjusted logistic regression.
